# Report from the Case-Book of Thos. S. Powell, M. D. Sparta, Georgia

**Published:** 1857-02

**Authors:** 


### ARTICLE IV.

### *Report from the Case-Book of* THOS. S. POWELL, M. D., *Sparta, Georgia.*

DRS. LOGAN & WESTMORELAND—I am aware that the utility of publishing isolated cases of disease has been much questioned, especially by some of our city *literati* of the profession. But while I would not say aught against those who have the time, talents and industry to give publicity to elaborate and scientific articles, I must say, that the archives of medical science would not be so replete with useful and practical knowledge, were it not for the many detached facts and observations found in the report of cases as they occur in general practice.

I will offer no further apology for placing a brief report of several cases of Pneumonia, from my Case-Book, at your disposal, to be published or not, at your discretion.

CASE 1. A large, robust negro boy, 18 years of age, had been indisposed for several days, with simple catarrh; on the 5th November, was exposed to the wet and cold, picking cotton; was attacked next morning about ten o'clock, with a chill; took his bed at night; fever very high; cough dry and distressing. His master gave him three Moffat's pills. Seven o'clock, A.M. Cough no better; fever supposed to be not quite so high; pills had operated several times. Three o'clock, P. M. Worse; more fever; cough more troublesome. I was sent for; saw him at 6 o'clock. Condition—suffering with severe headache; conjunctiva highly injected; tumid; skin hot and dry; pulse 120 per minute, full and bounding; cough almost incessant; expectoration tenacious, and much streaked with blood. Left syr. prun. virg., and ordered one tablespoonful to be given every two hours, counting the pulse at the time, and if above 90, to add 4 drops of veratrum viride. Flax seed infusion to be drank *ad libitum.*

Eight o'clock, A. M. Cough not so troublesome; expectorating more freely, and less blood; pulse 104; had been decreasing since 2 o'clock; gave 20 grains quinine and 30 drops of laudanum at once; ordered the syr. and veratrum to be continued, with the same proviso: by 11 o'clock, A. M., patient much improved; no pain; cough not so troublesome; pulse 90; skin soft and moist; gave no more quinine; directed the syr. prun. virg. to be continued until the cough subsided; adding 4 drops of veratrum when the pulse exceeded 80 or 85 per minute.

In a few days Mr. L. came to town and informed me that Isaac was rapidly recovering; had not taken any veratrum since the day after I saw him. He is now able to walk about the yard; coughs a little; sent him another bottle of the syrup.

Case 2. On the 8th of November, I was called to see Ellen, a negro woman, belonging to Wm. L., aged 22, stout and well made. Saw her at 2 o'clock, P. M. Her situation was as follows: Pulse 136, and small; skin dry; respiration frequent and laborious; unable to turn herself in bed; nervous system perfectly prostrated; tongue coated, but moist; cough frequent, dry and hard; ordered two tablespoonsful of the syr. prun. virg. with three drops of veratrum every two hours; the latter to be omitted after the pulse was reduced to 100 per minute.

9th—7 o'clock, P. M. Patient passed a restless night; pulse, the first part of the night, as high as 160; pulse now 130; no perceptible amendment, except that the pulse and skin are a little softer. Gave 20 grains quinine and 20 gtt. tinct. opii. at once, in half cup of warm sage tea. The syr. and veratrum to be continued.

10th—10 o'clock, A. M. Patient very much improved in every respect; pulse 72; respiration easy; skin moist; cough loose, and expectorating freely; gave no more quinine; ordered the syr. to be continued, with perfect rest and a soup diet.

12th—P. M. Much improved; pulse had not exceeded 80 since my last visit. Advised the syr. to be continued until the cough subsided. In a week Ellen was well.

Case 3. On the 9th of October, I was requested by Mr. W. to call and see a little negro girl about 10 years of age; saw her at 3 o'clock, P. M.; found her with a high fever; intense pain in the right side and chest; pulse 156; respiration quick; skin dry; cough hard; expectoration bloody. She had also been purged with Moffat's pills, as all are who dare complain in that neighborhood. Ordered one teaspoonful of the syr. prun. virg., with one drop of veratrum, every two hours, as long as the pulse exceeded 100 per minute. Flax seed infusion to be drank *ad libitum.*

10th—8 o'clock. Patient better in some respects; pulse the same, 156; skin much softer; respiration easier; cough not so frequent. Gave 15 grs. quinine and 10 gtt. tinct. opii. in half cup of warm sage tea at once; syrup and veratrum to be continued as before. 11th—11 o'clock, A. M. Much improved; pulse 95, and soft; skin moist. Gave no more quinine; ordered the syrup to be continued for several days, adding the veratrum every four hours, as long as the pulse exceeded 95 per minute. She recovered rapidly.

CASE 4. Bob, an old negro, 50 years of age, informed his master on the 16th of January last, that he was sick, and had been very unwell for a week; complained of headache, and a severe pain in the right side, and in the mammary region of the chest, particularly when he drew a deep inspiration or coughed. His master gave him a dose of oil, and ordered him to live light and remain in doors a few days. On the 22d, I was sent for; found him with high fever; complaining of great pain and oppression in the chest; respiration short and quick; cough frequent; expectoration viscid and rusty colored. Gave calomel 5 grs., Dover powders 5 grs., and ordered one tablespoonful of the syr. prun. virg., to be given every two hours; and 4 drops of veratrum viride to be added as long as the pulse exceeded 100 per minute. 23d—8 o'clock, A. M. No perceptible change, except the skin was softer. Gave 20 grs. quinine in half cup of warm sage tea at once, and ordered the previous treatment to be continued. 24th—8 o'clock. Less fever; some amendment in all of the symptoms; perspired freely the evening before; spent a more comfortable night; repeated quinine; continued the syrup and veratrum. 25th—10 o'clock. Bob is much improved; pulse 90 per minute. Gave 10 grs. quinine, and ordered the syrup to be continued for several days, and discontinue the veratrum. In ten or twelve days, Bob was well.

REMARKS.—I have the notes of a number of other cases similar to the above, but presume it unnecessary to detail them, all of which were successfully treated with quinine. I do not recommend it as the *sine qua non* in every climate, and in all the numerous varieties of this disease, but as a valuable auxiliary to other remedies in all cases in malarious districts, particularly when the fever manifests the least tendency to a remittent type.

